# Root Canal Cleaning after Different Irrigation Techniques: An Ex Vivo Analysis

**DOI:** 10.3390/medicina58020193

**Published:** 2022-01-27

**Authors:** Federica Di Spirito, Massimo Pisano, Mario Caggiano, Prashant Bhasin, Roberto Lo Giudice, Dina Abdellatif

**Affiliations:** 1 Department of Medicine, Surgery and Dentistry, Schola Medica Salernitana, University of Salerno, Via S. Allende, 84081 Baronissi, SA, Italy; pisano.studio@virgilio.it (M.P.); macaggiano@unisa.it (M.C.); 2 Department of Conservative Dentistry & Endodontics, Sudha Rustagi College of Dental Sciences and Research Faridabad, Faridabad 121002, India; drprashant.bhasin@gmail.com; 3 Department of Clinical and Experimental Medicine, University of Messina, 98122 Messina, ME, Italy; roberto.logiudice@unime.it; 4 Department of Endodontics, Faculty of Dentistry, University of Alexandria, Alexandria 21545, Egypt; dinaabdellatif81@gmail.com

**Keywords:** cleaning, endodontics, histology, internal heating, ultrasonic activation

## Abstract

*Background and Objectives*: The endodontic space is a complex area on both micro and macro levels; therefore, traditional irrigation techniques may not guarantee a complete cleaning of such a complicated tridimensional system. The presented ex vivo study aimed to evaluate root canal cleanliness, obtained through an equal volume of traditionally applied sodium hypochlorite (NaOCl), compared to ultrasonically activated NaOCl and ultrasonically activated NaOCl that had undergone intracanal heating NaOCl. *Materials and Methods*: A total of 60 freshly extracted human mandibular premolars underwent root sample length standardization (18 mm), root canal preparation and, based on the irrigation method employed, were randomly and equally assigned to three study groups, composed of root samples treated with ultrasonically activated NaOCl, ultrasonically activated NaOCl that had undergone intracanal heating and traditionally applied NaOCl. The root specimens were subsequently fixated with 4% buffered formalin solution and decalcified in Morse liquid. A total often 6-micron-thick serial cross-sections were obtained, dyed using hematoxylin and eosin and examined through an optical microscope at 40×, 100×, and 200×. *Results*: Ultrasonically activated NaOCl that had undergone intracanal heating showed a significantly smaller amount of debris compared to ultrasonically activated and traditionally applied NaOCl groups (*p* value < 0.05). *Conclusions*: Root canal cleanliness saw significant enhancements by ultrasonically activated NaOCl that had undergone intracanal heating.

## 1. Introduction

When teeth are diagnosed with inflamed vital pulps or infected with necrotic pulp tissues, the major aim of chemo-mechanical preparation is to target dissolving pulp tissue and dysrupt microbial biofilm. In particular, disinfection procedure should not only be confined to the major root canal space, but also reach the attached lateral canal system [1,2,3,4]. A complex lateral system like this includes macro-anatomies, in the form of lateral canals, isthmus, loop, delta and ramifications, as well as micro-anatomy, as the dentinal tubules. Accordingly, root canal irrigation, performed with a common syringe and needle, may not be able to produce the required shear stress, nor permit proper irrigant infiltration into lateral macro- and micro-anatomies [5], thus indirectly determining the persistence of residual pulp tissue or biofilm and eventually leading to persistent infections or re-infections [6,7] and to endodontic therapy failure in the complex lateral system.

Consequently, in view of subduing such shortcomings, endodontic research has focused, in recent years, on developing strategies to activate irrigation, to which end several techniques, including subsonic, sonic, ultrasonic, laser and manual dynamic ones, have been tested with no definitive results [5,8,9]. Such contrasting results may be partially attributable to the heterogeneity of canal mechanical preparation systems, length of irrigant activation and outcome of measurement processing among the studies.

The use of extra-orally heated canal irrigants, particularly sodium hypochlorite (NaOCl), is a well-known strategy for boosting pulp tissue solving effect [10,11]. However, the required buffering temperature may render this technique less effective in clinical use compared to in vitro applications. In order to overcome this setback, an alternative approach, specifically internal heating, was developed, allowing sodium hypochlorite to be heated, at controlled temperatures, within root canals, using heat carrier tips [12]. Notably, recent studies have shown that intracanal heating of NaOCl results in a drastic reduction in pulp tissue and microbial biofilm debris persistence onthe treated root canal walls when compared to irrigation with pre-heated NaOCl [13,14].

Given these considerations, the current ex vivo study aimed to evaluate, at a microscopic level, in conservatively shaped root canals, root canal cleanliness obtained through an equal volume of traditionally applied NaOCl, compared to ultrasonically activated NaOCl and ultrasonically activated NaOCl that had undergone intracanal heating. The null hypothesis was that there was no significant difference among the three irrigation techniques applied.

## 2. Materials and Methods

The present study was approved by the Institutional Review Board and the local Ethics Committee. Written informed consent forms were gathered from all of the participants.

### 2.1. Specimen Collection

Sixty human mandibular premolars, freshly extracted as part of the orthodontic therapy plan, underwent periodontal tissues detachment, using a curette, immediately after tooth extraction, and were collected in separate vials, containing 5 mL of 10% formalin solution. The validity of the experimental design was expressed beforehand.

### 2.2. Root Canal Preparation

The collected teeth were slitted at the level of the cemento-enamel junction to create root samples of a standardized length (18 mm). A K-file 10 mm size (Hyflex, Coltene/Whaldedent, Altstatten, Switzerland) was placed in each root canal until it was seen via the apex, and the working length was determined by deducting 0.5 mm from this measurement. Nickel-titanium rotary instruments, specifically 10/0.05 and 20/0.05 file instruments (Hyflex EDM, Coltene/Whaldedent, Altstatten, Switzerland), were exclusively employed on the entire working length to deliberately establish a conservative shaping of the root canals. Throughout the entire canal shaping, irrigation was performed with a total of 5 mL of 3% NaOCl, via a 30G needle in a disposable syringe, renewed every minute, for each root canal. Subsequently, root canals were rinsed with sterile saline, further irrigated with 3 mL of 17% EDTA for 1 min to eliminate the smear layer and lastly rinsed with 3 mL of sterile saline.

The 60 specimens were randomly and equally assigned to three groups (*n* = 20), composed of root samples treated with ultrasonically activated NaOCl (group A), ultrasonically activated NaOCl that had undergone intracanal heating (group B) and traditionally applied NaOCl (group C). The roots were tinted with nail varnish to avert irrigant extrusion. An equal volume of irrigant, 3% NaOCl (CanalProTM 3%, Coltene/Whaledent, Altstatten, Switzerland was injected into the root canals, using a 30G side-vented needle (CanalProColtene/Whaledent, Altstatten, Switzerland), at 2 mm from the working length, in the three groups.

For the ultrasonically activated NaOCl group (group A), NaOCl activation was performed through an ultrasonic file, connected to a cordless ultrasonic generator (Ultra smart ultrasonic activator, Coxo, Foshan City, China). The activation regimen comprehended eight cycles (20 s each), with 1 mL of irrigant freshened during each cycle. The ultrasonic tip was situated at 2 mm from the working length.

In ultrasonically activated NaOCl that had undergone intracanal heating (group B), ultrasonic activation was conducted as previously described and preceded by intracanal heating. The internal heating was performed for 8 s using a System-B heat source XF-tip (30/0.04) at 180 °C (Kavo Kerr, CA, USA) and was positioned at 3 mm from the working length.

The traditionally applied NaOCl group (group C) specimens were irrigated with 8 mL of NaOCl through a disposable syringe and needle at a 2 mL/min pace.

### 2.3. Evaluation of Root Canal Walls Cleanliness

Thereafter, specimens were dried out and fixated, using 4% buffered formalin solution for 48 h and rinsed beneath running water for one hour. Then, samples were soaked in Morse liquid to reach decalcification for four weeks, with the solution being replenished every two days. Six micron-thick serial cross-sections were obtained from the roots, based on a previously applied protocol [15,16]. Ten serial sections, at 2–5 mm from the apex of the root canal, were dyed, using hematoxylin and eosin.

Subsequently, obtained sections were examined under an optical microscope (OptiKa TB 290, Optika, Turin, Italy), at 40×, 100×, and 200×, using the dedicated Otpika Vision Lite software. Two independent, blinded, calibrated operators graded the quantity of pulp tissue debris in each section, based on the following criteria: grade 1—detectable debris on 75–100% of the area; grade 2—debris on 50–74% of the entire area; grade 3—debris on 25–49% of the entire area; grade 4—debris <24% throughout the area. In case of disagreement between investigators, a third one evaluated the samples; final grading was accomplished by discussion among investigators.

### 2.4. Data Presentation and Statistical Analysis

Frequency distribution of persisting pulp tissue and microbial biofilm debris was presented as lambda scores. Non-parametric tests were used for multiple comparisons among groups (Kruskal–Wallis). Additionally, Mann–Whitney test was employed for comparisons between pairs of groups.

## 3. Results

Ultrasonically activated NaOCl that had undergone intracanal heating (group B) manifested no or <25% debris for all of the sections (lambda 0.025), whereas the ultrasonically activated NaOCl group (group A) showed debris >25% in multiple sections and >50% in the remaining ones (lambda 0.0001). The traditionally applied NaOCl group (group C) revealed debris >50% in all of the sections.

**Figure 1:**
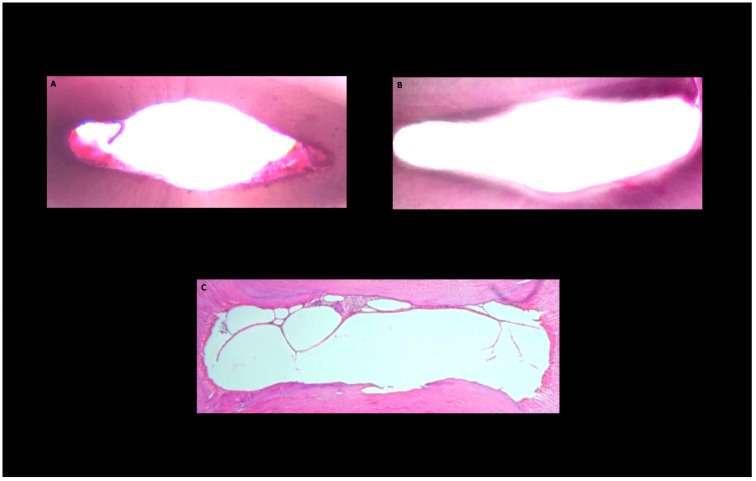
Histological images of the cross-sections obtained from the middle third of the root canal area after root canal preparation. Pulp tissue and microbial biofilm debris persisting and root canal wall protruding into the canal lumen (white) in ultrasonically activated (group **A**), ultrasonically activated preceded by intracanal heating (group **B**) and traditionally applied (group **C**) NaOCl groups.

**Figure 2:**
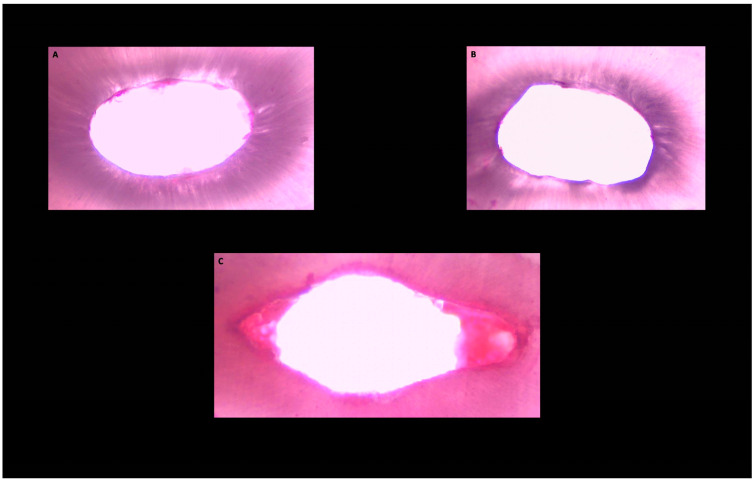
Histological images of the of the cross-sections obtained from the apical third of the root canal area after root canal preparation. Pulp tissue and microbial biofilm debris persisting and root canal wall protruding into the canal lumen (white) in ultrasonically activated (group **A**), ultrasonically activated preceded by intracanal heating (group **B**) and traditionally applied (group **C**) NaOCl groups.

Ultrasonically activated irrigation preceded by intracanal heating resulted in significantly cleaner canals (*p* value < 0.05) compared to ultrasonically activated irrigation alone and to syringe and needle irrigation (Figure 1 and Figure 2).

## 4. Discussion

The main purpose of endodontic treatment is the removal, as complete as possible, of damaged pulp tissues and microbial biofilm from the complex endodontic system [14,15]. Considering that the root canal system comprises the macroscopic anatomy of the major canal and lateral ones, along with ramifications, loops, isthmuses, deltas, as well as the so-called microscopic anatomies, as the dentinal tubules, for effective endodontic cleaning, irrigants should be able to penetrate such complicated root anatomies [5].

Ordinarily, bacteria can survive either as independent free-floating cells, in planktonic state, or as members of colonized surface-attached microbial communities, enclosed in a self-produced extracellular matrix that connects cells, overall identified as biofilm [17]. Biofilm bacteria usually shows a greater resistance to antimicrobial agents, up to 1000-fold higher, compared to that reported for the same microorganisms in fluid suspension [17,18]. Currently, the most common and effective strategy for counteracting biofilm in root canals consists of mechanical instrumentation and irrigant activation, leading to biofilm elimination [17].

It is well established that contemporary mechanical preparation strategies are not able to adequately reach root canal walls, thus leaving residual tissue and microbial residues inside the root canal system [19,20,21,22,23,24], and, consequently, negatively affect endodontic treatment outcomes, secondary to potential persistent infection and re-infection [17]. Therefore, it appears essential to improve irrigation procedure to subdue the inadequacy of current instrumentation techniques.

Irrigation efficacy can be improved by employing activated irrigants; for instance, an easy way to activate NaOCl is its pre-heating to a temperature of 50 °C. Pre-heated NaOCl solution has been proved to have higher antimicrobial effect and tissue dissolving capabilities [5,12,13]. Nevertheless, injecting pre-heated NaOCl into the root canal also has its constraints. The human body, in fact, can rapidly buffer NaOCl temperature, thusreducing the acquired heat empowered efficacy benefits [13]. Accordingly, it may be more convenient to heat NaOCl irrigant straight inside the root canal, by the means of heat carriers. Correspondingly, previous studies have highlighted that intracanal heating of NaOCl irrigant is more effective in eliminating residual organic tissues and removing hard tissue remnants from the major root canal area, when compared to pre-heated and non-heated NaOCl [14]. Irrigant activation, which can be obtained via sonic, ultrasonic, internal heating, or laser tools, has proved a remarkable enhancement in cleansing and disinfection of the endodontic system, being, therefore, considered an essential step in nonsurgical endodontic treatment. In accordance with such findings, the present ex vivo study outlined that ultrasonic activation subsequent to intracanal heating of NaOCl leads to significantly less remaining debris compared to ultrasonic irrigation of non-heated NaOCl.

## 5. Conclusions

The endodontic space is a complex area, on both micro and macro levels; therefore, traditional irrigation techniques may not guarantee a complete cleaning of such a complicated tridimensional system. The irrigation technique employing ultrasonically activated NaOCl that has undergone intracanal heating may be considered easy to carry out in routine clinical procedures, as well as being economical, since it does not require specific equipment. Moreover, ultrasonically activated NaOCl that has undergone intracanal heating irrigation, through the reduction in NaOCl viscosity, and a consequent higher penetration into dentinal tubules, has been proved to enhance antimicrobial effect, as well as tissue dissolving capabilities [5,12,13], and can be performed with no restrictions regarding tooth anatomy orclinical situations. Furthermore, this irrigation technique may be considered highly effective, which is in agreement with other authors’ findings, since the technique as applied in this study highlighted that root canal cleanliness was significantly enhanced by ultrasonically activated NaOCl that had undergone intracanal heating irrigation, as compared to ultrasonically activated and traditionally applied NaOCl.

However, although, to the authors’ knowledge, the present study is the first to assess from an hystological point of view these complex lateral macro- and micro-anatomies, following ultrasonically activated NaOCl that has undergone intracanal heating irrigation, especially combined to conservative shaping, and to compare root canal walls’ cleanliness obtained via three different irrigation techniques, the small sample size means that if they are to strengthen such ex vivo observations, forthcoming investigations should compare the results obtained from the presented technique for a complex lateral canal system with those from other irrigant activation strategies, combined to conservative shaping, both in preclinical and in clinical settings.

## Data Availability

All the data are available from the corresponding author upon reasonable request.

## Abbreviations

NaOCl	sodium hypochlorite
Ml	milliliters
Mm	millimeters
EDTA	Ethylenediaminetetracetic acid
G	Gauge
H	Hours
C	Degree Celsius
